# Breakthroughs in the Pathological Mechanism and Comprehensive Management of Pulmonary Hypertension

**DOI:** 10.31083/RCM50716

**Published:** 2026-10-14

**Authors:** Keke Zhou, Yun Gong, Jinxiao Zhang, Doudou Chen, Yu Liu, Zhihao Xu

**Affiliations:** ^1^Department of Respiratory, The Fourth Affiliated Hospital of School of Medicine, and Zhejiang University School of Medicine, International Institutes of Medicine, Zhejiang University, 322000 Yiwu, Zhejiang, China

**Keywords:** pulmonary hypertension, pulmonary arterial hypertension, pathological mechanism, therapy

## Abstract

Pulmonary hypertension (PH) is a rare and life-threatening vascular disorder characterized by complex and multifactorial pathogenesis. Emerging evidence indicates that pulmonary vascular remodeling is driven by coordinated abnormalities in genetic susceptibility, epigenetic regulation, metabolic reprogramming, immune-inflammatory crosstalk, and DNA damage repair. Recent advances in multi-omics profiling, single-cell technologies, and high-resolution imaging have substantially improved the understanding of PH pathobiology and disease heterogeneity. Current therapeutic strategies mainly target the three classical vasoactive pathways to alleviate vasoconstriction and improve hemodynamics. Notably, (bone morphogenetic protein) BMP/transforming growth factor‑β1 (TGF-β) pathway-targeted therapy, represented by sotatercept, has initiated a new era of disease-modifying treatment aimed at reversing pulmonary vascular remodeling. In addition, emerging therapies targeting immune, hormonal, and metabolic pathways have demonstrated promising therapeutic potential. Despite significant progress in both basic and clinical research, major challenges remain, including precise phenotypic stratification, long-term therapeutic safety, and individualized treatment optimization. This review summarizes recent advances in the molecular mechanisms and comprehensive management of PH and highlights future directions for translational research and precision medicine.

## 1. Introduction

Pulmonary hypertension (PH) is a group of malignant vascular diseases characterized by progressively elevated pulmonary circulatory pressure and irreversible remodeling of pulmonary vascular structure and function. PH ultimately leads to right-sided heart failure and even death [1,2]. It is associated with high disability, high mortality, and low rates of early diagnosis [3,4]. According to the 2022 international guidelines for the diagnosis and classification of PH, the disease is classified into five groups. Group 1, pulmonary arterial hypertension (PAH); Group 3, PH associated with lung diseases and/or hypoxia; and Group 4, pulmonary hypertension associated with chronic obstructive pulmonary disease (PH-COPD), are classified as precapillary PH. Group 2, PH associated with left heart disease, is classified as postcapillary PH. Group 5 includes PH with unclear and/or multifactorial mechanisms [5]. PAH, the first major subgroup of PH, is defined as an abnormal hemodynamic and pathophysiological condition characterized by a mean pulmonary arterial pressure (mPAP) >20 mmHg, a pulmonary arterial wedge pressure (PAWP) ≤15 mmHg, and a pulmonary vascular resistance (PVR) >2 Wood units (WU) [5]. Over the past decades, research on PH has evolved from the early concept of “vasoconstriction–vasodilation imbalance” to a more comprehensive understanding involving pulmonary vascular structural and functional remodeling, together with complex interactions among inflammatory, immune, genetic, and metabolic factors. This complex pathological change makes the diagnosis and management of PH extremely challenging [6,7].

In recent years, with the rapid development of molecular biology, genomics, and advanced imaging techniques and artificial intelligence, we have gained a deeper understanding of the pathological mechanisms of PH and achieved many breakthroughs in diagnosis and treatment [8], for example, single-cell RNA sequencing and immunofluorescence staining have revealed the unique heterogeneity of pulmonary vascular endothelial cells and mesenchymal cells [9,10], while digital spatial transcriptomics provide molecular structural characteristics for PH vascular lesions [11]. Meanwhile, the identification of novel biomarkers has provided new tools for early diagnosis and precise classification of PH [12]. In clinical management, the widespread use of drugs targeting the three classical pathways, including endothelin, nitric oxide, and prostacyclin signaling, has significantly improved symptoms and exercise capacity [13,14]. However, these therapies have limited ability to reverse pulmonary vascular remodeling or reduce long-term mortality. Recently, disease-modifying therapies targeting Bone morphogenetic protein (BMP)/transforming growth factor-β1 (TGF-β) signaling imbalance, represented by sotatercept, have emerged [15,16]. These agents mark a strategic shift in PH treatment from “simple vasodilation” toward “reversal of vascular remodeling”. In addition, therapeutic strategies targeting vascular remodeling, inflammation, and metabolic dysregulation are attracting increasing attention as promising approaches [17,18]. Despite substantial advances in both basic research and clinical management, several critical challenges remain. These include pronounced disease heterogeneity, incomplete understanding of pathogenic mechanisms, uncertain long-term safety and beneficiary populations of emerging therapies, and the lack of established individualized treatment strategies. Based on recent high-quality evidence, this review systematically summarizes current advances in PH pathogenesis, diagnostic strategies, targeted therapies, and emerging therapeutic directions. The aim is to provide a comprehensive reference for translational research and optimized clinical management.

## 2. Research Progress on the Pathological Mechanisms of PH

The occurrence and development of PH is a complex pathological process involving multiple factors and multiple links, with the core being the structural and functional abnormalities of pulmonary vessels, namely pulmonary vascular remodeling, manifested as pulmonary vascular constriction, thickening of the vessel wall, narrowing and even occlusion of the vessel lumen, ultimately leading to adverse outcomes such as increased pulmonary vascular resistance and right-sided heart failure [19].

### 2.1 The Core Drivers of Pulmonary Vascular Remodeling and Multi-Cellular Synergy

#### 2.1.1 Endothelial Cell Dysfunction

Lung endothelial cells play a central role in this process, driving vascular remodeling by upregulating pro-inflammatory and endothelial-mesenchymal transition-related genes [20]. Zhou et al. [9] identified disease-related endothelial cell populations and key signaling pathways in patients with systemic sclerosis associated with PH and idiopathic pulmonary hypertension through single-cell RNA sequencing, and observed the presence of transitional cells, suggesting the role of the key pathological process of endothelial-mesenchymal transition (EndMT) in PH vascular lesions. In addition, high shear stress (HSS) was shown to induce EndMT in pulmonary artery endothelial cells and promote PAH by reducing ETS-related gene (ERG), a member of the E26 transformation-specific (ETS) family of transcription factors. and histone H3 lysine 27 acetylation, while ERG supplementation reversed this process [21]. Jiao et al. [22] also supported the key role of EndMT in PAH, finding that knockdown of *eukaryotic translation initiation factor 3 subunit A (eIF3a) *could reduce EndMT in endothelial cells by inhibiting the TGFβ1/SMAD (SMAD family member) signaling pathway, suggesting that eIF3a could become a new therapeutic target for PAH. These studies collectively highlighted the central role of endothelial cells in vascular remodeling and revealed a complex network of multiple regulatory EndMT, providing potential directions for targeted therapy.

#### 2.1.2 Smooth Muscle Cell Proliferation

In addition to endothelial cell alterations, abnormal proliferation and migration of pulmonary artery smooth muscle cells (PASMCs) are also important links in pulmonary vascular remodeling. Bordan et al. [23] discovered through microarray analysis and RNA sequencing techniques that *PDZ-binding kinase (PBK)* is upregulated in PAH and drives PASMC proliferation by promoting cell division, suggesting that inhibition of PBK or gene knockout may effectively reverse or prevent the occurrence and development of PH. In addition, a novel long non-coding RNA (lncRNA), vessel‑enriched lncRNA regulated by *platelet-derived growth factor-BB (PDGF-BB) (VELRP)*, was found upregulated in PASMCs of PAH patients and regulated PASMC proliferation through the *the WD repeat domain 5 (WDR5)/cyclin-dependent kinase (CDK)* signaling pathway.

Knockdown of *VELRP* improved right ventricular systolic pressure and vascular remodeling in rat PH models [24]. Ranasinghe et al. [25] revealed from an epigenetic perspective that the *sphingosine kinase 2 (SPHK2)*/sphingosine‑1‑phosphate (S1P) axis promotes PH by altering histone acetylation in smooth muscle cells. These studies elucidate the molecular mechanisms of abnormal proliferation of PASMCs at multiple levels, providing a theoretical basis for the development of targeted drugs against PASMCs.

#### 2.1.3 Extracellular Matrix Remodeling and Fibrosis

Abnormal deposition and remodeling of extracellular matrix (ECM) are key factors in pulmonary vascular fibrosis, especially more prominent in PH. A comprehensive analysis of proteoglycan expression and deposition in the pulmonary artery walls of healthy and PH patients revealed elevated expression, deposition, and synthesis of major vascular proteoglycans in the pulmonary arteries of idiopathic PAH (IPAH) and fibrosis-associated PH patients, and revealed that TGF-β regulates the expression of certain proteoglycans to promote vascular fibrosis [26]. Other studies further confirmed elevated TGF‑β1, phosphorylated SMAD family member 2/3 (p‑Smad2/3), SMAD family member 7 (Smad7), and β‑catenin in the pulmonary artery of patients with idiopathic pulmonary fibrosis, which drive EndMT and are closely associated with intimal thickening and elastin deposition [27]. In addition, the blocking of integrin α5β1 is considered a potential disease-modifying strategy for PAH as it reduces ECM deposition in PASMC, improves cardiac function, and lowers pulmonary artery wall thickness and fibrosis [28]. These studies together paint a complex picture of ECM remodeling in PH and point to therapeutic potential targeting the fibrotic pathway (Fig. 1).

**Fig. 1.**
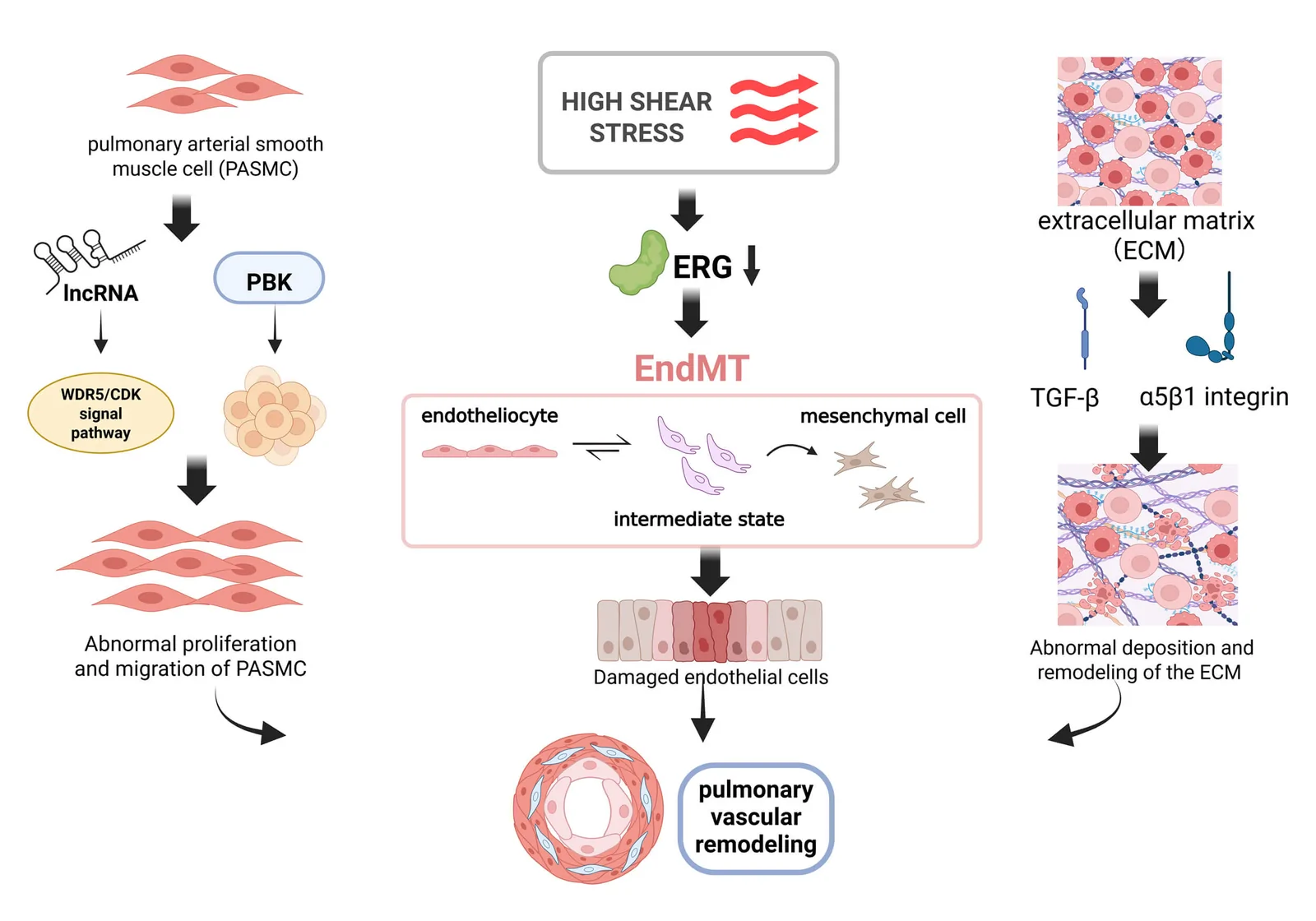
**Synergistic multicellular mechanisms driving pulmonary vascular remodeling**. Abbreviations: PASMC, pulmonary arterial smooth muscle cell; lncRNA, long non‑coding RNA; PBK, PDZ‑binding kinase; HSS, high shear stress; ERG, ETS‑related gene; EndMT, endothelial‑to‑mesenchymal transition; ECM, extracellular matrix; TGF‑β, transforming growth factor‑β; α5β1 integrin, integrin alpha‑5 beta‑1.

#### 2.1.4 Cellular Senescence

Cellular senescence is involved throughout multiple stages of PAH development and progression. Early senescence responses may participate in stress repair after injury, whereas late-stage senescence promotes irreversible pulmonary vascular remodeling [29]. In pulmonary artery endothelial cells from patients with congenital heart disease-associated PAH (CHD-PAH), senescence markers such as p16 and p21 are upregulated. These changes are accompanied by the formation of a senescence-associated secretory phenotype (SASP) [30], which sustains a pro-inflammatory, pro-proliferative, and pro-fibrotic microenvironment. Mechanistically, hypoxia can activate the mammalian target of rapamycin (mTOR)/ribosomal protein S6 kinase 1 (S6K1) pathway and induce senescence in PASMCs. Senescent PASMCs subsequently promote abnormal proliferation of neighboring PASMCs through paracrine secretion of interleukin‑6 (IL‑6), thereby forming a positive feedback loop linking senescence, inflammation, and proliferation [31]. At the same time, cellular senescence is closely coupled with other major pathogenic mechanisms in PAH, including *bone morphogenetic protein receptor type 2 (BMPR2)* deficiency, EndMT, immune inflammation, and mitochondrial metabolic dysfunction [32,33,34]. Importantly, senolytic strategies targeting senescent cells have been shown to improve vascular remodeling in experimental PAH models [30]. These findings suggest that cellular senescence is not only a consequence of PAH progression, but may also represent an important driver of persistent disease progression.

### 2.2 The Role of Inflammation and Immune Response in PH

#### 2.2.1 Crosstalk Between Immune Cells and Vascular Cells

A growing body of evidence has demonstrated prominent perivascular inflammatory responses in the lung tissues of patients with PAH [35,36]. Pulmonary vascular cells and multiple immune cell populations can jointly produce inflammatory mediators, thereby promoting vasoconstriction, abnormal cellular proliferation, and extracellular matrix deposition, ultimately leading to pulmonary vascular remodeling.

##### 2.2.1.1 Macrophages

Among immune cell populations, macrophages are important participants in the inflammatory response. Qiu et al. [37] found that MHC-IIhi LYVE1lo CCR2hi interstitial macrophages promote mesenchymal fibrosis in the pulmonary artery and lead to PH, while these macrophages promote fibroblast-like PASMC proliferation in the *wingless‑type MMTV integration site family member 11 (WNT11)*/planar cell polarity (PCP) pathway.

Abid et al. [38] also highlighted the role of C‑C chemokine receptor 2 (CCR2)/C‑C chemokine receptor 5 (CCR5)-mediated macrophage-smooth muscle cell crosstalk in PH. Studies have shown that *BMPR2*-deficient human pulmonary artery endothelial cells exhibit approximately twofold increased granulocyte-macrophage colony-stimulating factor (GM-CSF) expression following TNF stimulation [39]. GM-CSF not only recruits macrophages but also promotes the release of pro-inflammatory cytokines. Elevated GM-CSF levels have also been observed in IPAH patients [39], further supporting the critical role of the endothelial cell–macrophage inflammatory axis in PAH. Complex crosstalk between other immune cell populations and vascular structural cells also plays an important role in PH pathogenesis and progression [40].

##### 2.2.1.2 Regulatory T Cells (Tregs)

In contrast to macrophages, Tregs mainly exert immunosuppressive and vasculoprotective effects in PH. Taraseviciene-Stewart et al. [41] demonstrated that athymic nude rats lacking T cells developed severe pulmonary vascular lesions after SU5416 (semaxanib) exposure. Reconstitution with cluster of differentiation 4⁺ (CD4⁺), cluster of differentiation 25⁺ (CD25⁺), forkhead box P3⁺ (Foxp3⁺) Tregs markedly attenuated pulmonary vascular remodeling and pulmonary hypertension [42]. Subsequent studies further confirmed that Tregs play a dominant role in protecting female subjects against PH [43]. Tregs negatively regulate interleukin-18 (IL-18) secretion through intrinsic *NOD-like receptor family CARD domain-containing protein 3 (NLRC3)* signaling, thereby suppressing EndMT in pulmonary venous endothelial cells and attenuating pulmonary venous remodeling [44]. In addition, Treg-derived interleukin-35 (IL-35) inhibits endothelial cell proliferation through the *signal transducer and activator of transcription 1 (STAT1)* signaling pathway and reverses pathological pulmonary vascular remodeling [45]. Beyond endothelial cells, Tregs may also participate in PAH progression by suppressing abnormal PASMC proliferation and modulating fibroblast activation [46]. Importantly, bidirectional regulation exists between Tregs and vascular endothelial cells. Tregs maintain endothelial barrier integrity, reduce vascular permeability, and suppress endothelial inflammatory activation through immunomodulatory molecules such as interleukin-10, TGF-β, and cytotoxic T-lymphocyte-associated protein 4 (CTLA-4) [42,47]. Conversely, endothelial cells regulate Treg migration, localization, and immune homeostasis through adhesion molecules and chemokines [48]. This dynamic interaction [49] further highlights the importance of immune–vascular crosstalk in pulmonary vascular remodeling.

##### 2.2.1.3 Natural Killer (NK) Cells

In addition to Tregs, NK cells also contribute to the inflammatory microenvironment in PH. Studies have shown that mice with congenital NK cell deficiency spontaneously develop pulmonary hypertension [50], suggesting an essential role of NK cells in maintaining pulmonary vascular homeostasis. In the PAH microenvironment, TGF-β signaling suppresses NK cell function [51]. Therefore, targeting TGF-β signaling in NK cells has been proposed as a potential immunotherapeutic strategy. Furthermore, stress-induced *transcription factor 7 (TCF7)* activation enhances interactions between NK cells and PASMCs, leading to abnormal PASMC proliferation and phenotypic switching, thereby promoting medial thickening and luminal narrowing of pulmonary arteries [52]. Single-cell sequencing studies further demonstrated that T cells and NK cells highly express *C‑C motif chemokine ligand 5 (CCL5)* in the IPAH pulmonary vascular microenvironment. CCL5 specifically recruits and adheres to pulmonary artery endothelial cells and PASMCs, forming an abnormal cellular communication network [53]. These findings suggest that NK/T cell-mediated chemokine networks may represent an important link between immune inflammation and pulmonary vascular remodeling.

##### 2.2.1.4 Pulmonary Artery Adventitial Fibroblasts (PAAFs)

PAAFs have recently been recognized as important “signaling hubs” in PH-associated inflammation [54]. In PH patients, pulmonary fibroblasts establish a pro-inflammatory microenvironment, promote differentiation of T lymphocytes toward pro-inflammatory phenotypes, and reduce Treg abundance, thereby aggravating vascular inflammation and remodeling [55]. At the same time, fibroblasts secrete multiple inflammatory cytokines and chemokines that recruit macrophages and lymphocytes [56,57]. Through paracrine mechanisms, fibroblasts also promote PASMC proliferation, forming a sustained positive feedback loop linking inflammation and remodeling. This immune–vascular interaction spans multiple stages of PH pathogenesis and progression, providing an important foundation for the development of novel precision therapies targeting intercellular crosstalk.

#### 2.2.2 Immune-Inflammatory Microenvironment

Immune activation is considered an important driving factor of pulmonary vascular remodeling in PH. Clinical studies have demonstrated elevated circulating inflammatory cytokines in patients with idiopathic and heritable PAH, and these cytokine levels are associated with patient survival outcomes [58], suggesting that systemic inflammation contributes to disease progression. Within the local pulmonary vascular microenvironment, lung tissue from IPAH patients contains perivascular tertiary lymphoid structures accompanied by infiltration of B cells, T cells, dendritic cells, and plasma cells, as well as immunoglobulin deposition. These structures may locally generate pathogenic autoantibodies [36], thereby further promoting autoimmune injury and pulmonary vascular remodeling. Animal studies also support this concept: multiple experimental PH rat models have demonstrated that the mast cell–B cell axis and IL-6 signaling promote vascular remodeling in experimental pulmonary hypertension [59,60]. Collectively, these findings indicate that immune activation in PH is not merely a secondary phenomenon. Instead, it actively drives pulmonary vascular disease progression through systemic inflammation, local immune cell infiltration, autoantibody production, and cytokine-mediated signaling pathways.

### 2.3 Genetic and Epigenetic Mechanisms of PH

#### 2.3.1 *BMPR2* Is a Core Pathogenic Gene

Among all known PAH-associated genes, *BMPR2* is the most important pathogenic gene [61,62,63]. *BMPR2 *mutations are identified in approximately 70–80% of familial PAH cases and 10–20% of IPAH patients [64]. These pathogenic variants disrupt the BMPR2‑mediated TGF‑β superfamily signaling pathway. In PAH, mutations in *BMPR2 *and related genes lead to suppression of BMP-mothers against decapentaplegic homolog 1/5/8 (Smad1/5/8) signaling and relative enhancement of TGF-β-Smad2/3 signaling [65,66,67]. This signaling imbalance is considered a central mechanism underlying PAH pathogenesis. Loss of *BMPR2 *function or downregulation of BMPR2 signaling weakens anti-proliferative signaling pathways; this subsequently promotes abnormal proliferation of PASMCs [68], endothelial dysfunction [69,70], and EndMT [71]. These findings not only explain the molecular basis of heritable PAH, but also demonstrate that *BMPR2 *is not merely a susceptibility gene. Instead, it represents a central regulatory node linking endothelial dysfunction, vascular remodeling, and pathological proliferation.

#### 2.3.2 Other Key Pathogenic or Susceptibility Genes

Beyond *BMPR2*, multiple pathogenic and susceptibility genes associated with PH have been identified in recent years. A landmark study published in 2001 first established activin receptor-like kinase 1 (ALK1, encoded by the *activin A receptor like type 1 (ACVRL1)* gene) as a pathogenic gene in hereditary hemorrhagic telangiectasia-associated pulmonary arterial hypertension (HHT-PAH) [72]. Subsequent case studies further confirmed that loss-of-function mutations in *ACVRL1* directly contribute to HHT-PAH pathogenesis [73]. Currently, mutations in *ACVRL1* and Endoglin (encoded by the gene *endoglin (ENG)*) [74] are regarded as core targets in genetic testing for HHT-PAH. In addition, case reports describing rare sequence variants in BMP signaling mediators, including *SMAD1, SMAD4*, and *SMAD9*, have provided preliminary evidence supporting the involvement of SMAD family genes in PAH pathogenesis [75]. These findings further emphasize the central role of the TGF-β signaling pathway in PAH development. Other studies have demonstrated that *caveolin-1* (*CAV1*) [76], * ATPase phospholipid transporting 13A3 (ATP13A3)* [77,78], and *growth differentiation factor 2 (GDF2) *[77,79] contribute to the dysfunction of the* BMP *signaling pathway through distinct protein-encoding mechanisms. *SRY-box transcription factor 17 (SOX17)* is also critical for cardiovascular morphogenesis and arterial differentiation [80]. Wang et al. [81] reported a heterozygous *SOX17* mutation in a multigenerational family, resulting in impaired transcriptional activity of the downstream target gene *notch receptor 1 (NOTCH1).* Importantly, the NOTCH signaling pathway is a key regulator of arterial endothelial cell fate determination and vascular development. Furthermore, analyses of coding variants in *BMPR2*-negative patients identified heterozygous mutations in genes without direct effects on the TGF-β/BMP pathway, such as *potassium channel subfamily K member 3 (KCNK3) *[82], which encodes potassium channel subfamily K member 3. Pulmonary veno-occlusive disease (PVOD) and pulmonary capillary hemangiomatosis (PCH), a distinct form of PH with unique clinical and pathological characteristics, are caused by biallelic recessive mutations in *eukaryotic translation initiation factor 2 alpha kinase 4 (EIF2AK4) *[83,84]. These findings highlight the genetic heterogeneity and diverse inheritance patterns observed in PH. Overall, PH-associated genes with strong supporting evidence are mainly involved in biological processes including BMP/TGF-β signaling, transcriptional regulation, ion channel function, and endothelial homeostasis, reflecting the multidimensional complexity of the genetic mechanisms underlying PH.

#### 2.3.3 DNA Damage and Repair Abnormalities

Increasing evidence suggests that DNA damage and defective DNA repair are not merely manifestations of cellular stress in PH, but also represent important pathogenic mechanisms directly involved in pulmonary vascular remodeling. Among these mechanisms, abnormal activation of the DNA damage/poly(ADP‑ribose) polymerase 1 (PARP‑1) signaling axis is considered a central driver of PAH development and progression. PARP inhibitors targeting this pathway have been shown to reverse disease progression in experimental PH rat models [85], indicating that the DNA damage response (DDR) is not only a pathological marker, but also a functionally relevant therapeutic target. In addition, loss of *BMPR2* signaling has been closely linked to dysfunction of DNA repair networks [86]. *BMPR2* deficiency leads to persistent accumulation of unrepaired DNA damage and further downregulates expression of the transcription factor *forkhead box F1 (FOXF1)*. Restoration of *FOXF1* expression not only repairs DNA damage but also reverses the PH phenotype [87]. These findings suggest that the *BMPR2–FOXF1* axis may represent an important molecular pathway linking genetic susceptibility to impaired DNA repair. Beyond *BMPR2*-related mechanisms, the Fanconi anemia DNA repair pathway has also been implicated in PH pathogenesis; deficiency of the core repair factor *Fanconi anemia complementation group L (FANCL)* impairs the ability of pulmonary vascular endothelial cells to repair DNA damage [88]. Mitochondrial DNA (mt DNA) damage has also attracted increasing attention: under hypoxia–reoxygenation conditions, *BMPR2*-deficient endothelial cells are prone to mt DNA deletion and mitochondrial dysfunction, ultimately leading to persistent PH development [32]. Overall, DNA damage response-related pathways, including key molecules such as PARP*, FOXF1*, and *FANCL*, may represent promising novel therapeutic targets for PH.

#### 2.3.4 Genetic Differences Between Different Populations

##### 2.3.4.1 Children and Adults

Studies have shown that childhood-onset PAH carries a substantially greater genetic burden than adult-onset PAH. Genetic factors account for approximately 35% of pediatric IPAH cases, compared with only about 21% in adult IPAH [89,90].

In pediatric PAH, de novo variants, which are not inherited from parents, make a significant contribution to disease development and are estimated to account for approximately 15% of cases [79]. In contrast, such variants are less common in adult PAH. Another study further supported these findings by demonstrating an approximately twofold enrichment of deleterious de novo variants in pediatric IPAH patients without known risk gene mutations [91]. Therefore, the genetic architecture of pediatric PAH is more strongly influenced by de novo mutations. In adult-onset PAH, the prevalence in females is approximately three to four times higher than in males. However, this sex predominance is not observed in pediatric PAH [89,92,93], suggesting a lower dependence on sex-specific modifying factors during childhood. In addition, pediatric PAH is more commonly associated with congenital heart disease and developmental lung disorders [92,94,95]. These phenotypic associations are closely linked to genetic background and reflect the complex interaction between genetic and environmental factors in pediatric populations. Array-based comparative genomic hybridization (aCGH) and sequencing studies in childhood-onset PAH identified deletions and loss-of-function mutations in *T‑box transcription factor 4 (TBX4)*, a gene also associated with small patella syndrome [96]. *TBX4 *mutations are now recognized as one of the most common genetic causes of pediatric PAH. In pediatric IPAH/familial PAH patients, in addition to the most common* BMPR2* mutations (detection rate approximately 12.5%), mutations in *ACVRL1* (approximately 10%) and *TBX4* (approximately 7.5%) occur at significantly higher frequencies than in adult PAH patients [89,97]. Overall, the genetic landscape of PAH differs substantially between pediatric and adult populations.

##### 2.3.4.2 Sex Differences

Data from multiple international registries consistently show that the proportion of female PAH patients is approximately 2‑ to 4‑fold higher than that of males [98,99]. Studies have suggested that this sex bias is not only related to sex hormones, but also involves genetic mechanisms such as abnormal X-chromosome inactivation and the protective effects of the Y chromosome [100]. Although females are more susceptible to PAH, male patients generally have worse clinical outcomes [101,102]. The physiological basis of this “sex paradox” is highly complex and remains incompletely understood. Regarding genetic susceptibility, a meta-analysis demonstrated that the prevalence of *BMPR2* mutations is approximately 34.8% in male PAH patients, compared with 29.4% in females; moreover, among PAH patients carrying *BMPR2 *mutations, males exhibit a higher risk of death or lung transplantation [103]. Sex hormones and their metabolic pathways also play critical roles in these sex differences. For instance, estrogen and its receptors modulate the BMP/TGF‑β signaling pathway [104]; dysregulation of estrogen metabolism leads to accumulation of harmful metabolites such as 16α‑hydroxyestrone [105]; alterations in sex hormone profiles in postmenopausal women are also involved [106]. These interactions between sex hormones and genetic factors may partially explain the higher prevalence of PAH in females. Furthermore, sex differences are also reflected in right ventricular adaptation [107,108], immune responses [43,109], and molecular mechanisms involving gene–hormone interactions. Therefore, sex must be considered a critical variable in studies of PAH genetics and in the design of precision therapeutic strategies.

#### 2.3.5 Epigenetic Regulation Is an Important Complementary

Epigenetic alterations also play important roles in the pathogenesis of PH. In heritable pulmonary arterial hypertension (HPAH) patients without detectable *BMPR2* mutations, abnormal hypermethylation of the *BMPR2* promoter region suppresses *BMPR2* transcription, leading to functional downregulation of *BMPR2* signaling [110]. These findings provide a new explanation for the molecular basis of HPAH in patients lacking identifiable *BMPR2 *mutations. *TET methylcytosine dioxygenase 2 (TET2)*, a key enzyme involved in DNA demethylation, is downregulated in the majority of PAH patients (>86%) [111]. Loss-of-function mutations in *TET2* are associated with clonal hematopoiesis, vascular inflammation, and adverse vascular remodeling. Histone deacetylases (HDACs) regulate the acetylation status and activity of cytoplasmic proteins; increased HDAC activity promotes vascular inflammation and cellular proliferation [112]. HDAC1, HDAC5, and HDAC6 have been shown to be upregulated in the lungs of PAH patients [113]; valproic acid (VPA), as an HDAC inhibitor, has exhibited anti-remodeling and disease-reversing effects in experimental PAH models [112,114], supporting the therapeutic potential of HDAC inhibition in PAH. MicroRNAs are also closely involved in PAH pathogenesis. Downregulation of microRNA-204 (miR-204) [115] and upregulation of microRNA- 210 (miR-210) [116] together represent key molecular events driving PASMC phenotypic switching and pulmonary vascular remodeling. In monocrotaline (MCT)-induced PAH mouse models, the natural compound plumbagin restored *BMPR2* expression by suppressing microRNA‑21‑5p (miR‑21‑5p) [117], reversed PASMC phenotypic transition, and attenuated pulmonary vascular remodeling. In addition, microRNA-138-5p (miR‑138‑5p) has been found to be upregulated in both PH patients and experimental PH rat models. Inhibition of miR-138-5p in rat models restored *KCNK3 *expression, improved pulmonary hemodynamics, and reduced pulmonary vascular remodeling [118].

#### 2.3.6 Non-Coding RNA System

Non-coding RNA networks participate in pulmonary vascular remodeling in PAH through post-transcriptional regulation and competing endogenous RNA (ceRNA) mechanisms. MiR-204 is significantly downregulated in PASMCs from PAH patients, and its reduced expression is associated with disease severity [115]. Reduced miR‑124 promotes aerobic glycolysis and hyperproliferation of pulmonary artery endothelial cells via PTBP1 (polypyrimidine tract‑binding protein 1)‑mediated PKM (pyruvate kinase M) splicing in PAH [119]. In addition, the long non-coding RNA (lncRNA) MALAT1 regulates PASMC proliferation and migration through ceRNA mechanisms [120], further supporting the involvement of non-coding RNA networks, including miRNA, lncRNA, and circular RNA (circRNA), in PAH vascular remodeling. CircRNA can also regulate apoptosis, proliferation, migration, and ion channel function in PAH-related cells [121]. These findings highlight the multilayered regulatory role of non-coding RNA in pulmonary vascular remodeling.

#### 2.3.7 RNA Modification and RNA Editing

RNA modification and RNA editing have recently emerged as rapidly developing research areas in PH. Among these mechanisms, N6-methyladenosine (m6A) RNA modification exerts cell type-dependent bidirectional regulatory effects in PH by targeting different genes, such as* GRB2-related adaptor protein (GRAP)* in PASMCs [122] and *Kruppel-like factor 2 (KLF2)* in endothelial cells [123]. Through these mechanisms, m6A modification regulates smooth muscle proliferation and EndMT, respectively. At the same time, RNA editing also plays an important protective role. RNA editing mediated by adenosine deaminase acting on *RNA 1 (ADAR1)*, which is specifically expressed in smooth muscle cells, has been shown to suppress excessive activation of innate immunity. Studies by Kim et al. [124] and Woodcock et al. [125] collectively demonstrated that *ADAR1* deficiency enhances interferon-related inflammatory signaling, thereby promoting abnormal activation of pulmonary vascular cells and accelerating PH progression. These findings reveal the multidimensional role of epitranscriptional regulation in PH pathogenesis.

The pathogenic paradigm of PH has evolved from a simple imbalance between vasoconstriction and vasodilation into a highly complex network involving genetic susceptibility, epigenetic regulation, metabolic reprogramming, immune-inflammatory crosstalk, defective DNA damage repair, and cellular senescence. These findings emphasize that PH is not a single-gene disorder, but rather a complex syndrome driven by interactions among multiple genes, epigenetic mechanisms, and dysregulated cellular networks. In the future, patient stratification based on genotype and molecular phenotype will become a major direction for precision medicine in PH.

### 2.4 Metabolic Abnormalities and Mitochondrial Damage Mechanisms

PH is characterized by activation of proliferative and anti-apoptotic signaling pathways, as well as metabolic and mitochondrial dysfunction, in multiple cell types including endothelial cells [126].

#### 2.4.1 Metabolic Reprogramming

Recent studies have shown that pulmonary vascular cells in PH exhibit tumor-like metabolic features known as the “Warburg effect”. This phenomenon was initially described in cancer cells and is characterized by a metabolic preference for glycolysis even under oxygen-sufficient conditions [127]. Aerobic glycolysis supports rapidly proliferating cells by meeting increased metabolic demands while simultaneously providing metabolic intermediates for nucleotide and lipid synthesis. Endothelial cells possess strong glycolytic capacity and produce large amounts of lactate even under high oxygen tension [128]. Glycolytic activity varies among different endothelial cell subtypes. Arterial endothelial cells are relatively more oxidative, whereas pulmonary microvascular endothelial cells (PMVECs) display a more glycolytic and proliferative phenotype [129,130]. This metabolic shift provides rapid energy production and biosynthetic substrates, thereby supporting the abnormal proliferation and apoptosis resistance of pulmonary artery smooth muscle cells and endothelial cells.

#### 2.4.2 Mitochondrial Dysfunction

Pulmonary arterial cells from patients with PH and experimental animal models exhibit profound mitochondrial metabolic dysfunction [131]. These abnormalities include impaired oxidative phosphorylation through the electron transport chain (ETC) and disruption of tricarboxylic acid (TCA) cycle homeostasis [132]. Such alterations not only reduce ATP production, but also promote a metabolic shift toward glycolysis-dependent energy generation, thereby supporting a rapidly proliferative phenotype. Although reactive oxygen species (ROS) play essential physiological roles, including signal transduction and maintenance of vascular tone, excessive ROS production is harmful to cellular and organ function [133,134]. Mitochondria are both a major source and a major target of ROS [135]. Consequently, mitochondrial dysfunction markedly increases oxidative stress and leads to excessive ROS accumulation. Elevated ROS levels activate pro-proliferative signaling pathways and suppress apoptosis through regulation of dynamin-related protein 1 (Drp1), thereby enhancing the survival of pulmonary artery endothelial cells and smooth muscle cells [136,137,138]. This represents a characteristic anti-apoptotic feature of pulmonary vascular remodeling. In addition, alterations in mitochondrial dynamics contribute to PH pathogenesis. Imbalance between mitochondrial fission and fusion results in mitochondrial fragmentation and functional impairment [139,140]. These structural abnormalities directly promote intracellular ROS generation and metabolic reprogramming. For example, reduced expression of mitofusin-2 (MFN2) [141] or increased activation of DRP1 [142] has been observed in PH patients and rat PH models. These changes increase mitochondrial fission and produce fragmented mitochondrial morphology, thereby promoting PASMC proliferation and reducing apoptosis, ultimately contributing to pulmonary vascular remodeling. Mitochondrial injury also disrupts Ca^2+^ homeostasis and intracellular signaling integration [143]. By impairing calcium regulation and apoptotic pathways, mitochondrial dysfunction further sustains proliferative and anti-apoptotic phenotypes. Dysfunction of the mitochondrial calcium uniporter (MCU) reduces mitochondrial matrix calcium concentration, thereby suppressing pyruvate dehydrogenase activity and promoting aerobic glycolysis [144]. These changes enhance PASMC proliferation and apoptosis resistance. At the same time, increased cytosolic free calcium activates the calcineurin–nuclear factor of activated T-cells (NFAT) signaling pathway [145], further driving excessive cellular proliferation.

#### 2.4.3 Lysosome Biology and the Metabolic Inflammation Axis

Lysosomal biology and the metabolic–inflammatory axis have also gradually emerged as important areas. In PAH, deficiency of nuclear receptor coactivator 7 (NCOA7) induces lysosomal dysfunction, which subsequently triggers oxysterol and bile acid metabolic reprogramming [146], thereby promoting endothelial inflammation and vascular remodeling. *BMPR2*, a key protective receptor in PAH, can be degraded through autophagy-associated lysosomal pathways, resulting in insufficient BMP signaling [147]. *BMPR2* heterozygous mutations and inflammation can further synergistically enhance autophagy, thereby forming a vicious cycle. Further studies have demonstrated that the lysosomal protease cathepsin L participates in vascular remodeling by promoting *BMPR2*-associated pyroptotic signaling pathways [148]. Lysosomes are not merely intracellular degradation systems, but may also represent critical hubs linking metabolic abnormalities, inflammation, *BMPR2* signaling imbalance, and pulmonary vascular remodeling.

Overall, the development and progression of PH result from complex interactions among multiple pathological mechanisms. Metabolic dysfunction, oxidative stress, dysregulated cell death, and inflammatory responses are closely interconnected, forming a highly complex pathogenic network.

## 3. Diagnostic and Prognostic Assessment Strategies for PH

Clinically, the early symptoms of PH are often vague and non-specific, and early diagnosis can bring survival advantages. Therefore, early diagnosis of the disease and accurate prognosis assessment are crucial for improving patient outcomes [149,150]. With technological advancements, hemodynamic assessment, imaging techniques and biomarkers are playing an increasingly important role in this field.

### 3.1 Multi-Dimensional Omics and Novel Biomarkers

In recent years, research on biomarkers for PH has gradually shifted from traditional single indicators toward multidimensional integration, including proteomics, metabolomics, transcriptomics, and single-cell omics. These technologies analyze disease mechanisms from molecular to cellular levels and provide new candidate biomarkers for diagnosis and prognostic assessment.

#### 3.1.1 Proteomics

In the field of proteomics, studies have identified a prognostic model composed of nine plasma proteins through large-scale plasma protein screening [151]. This model significantly improves mortality risk prediction beyond traditional indicators such as N‑terminal pro‑B‑type natriuretic peptide (NT‑proBNP), highlighting the value of circulating proteomics in risk stratification. Research has found that inflammation-related circulating proteins are closely associated with PH patient outcomes [152,153], supporting the critical role of inflammatory responses in disease progression. Recent studies have further identified multiple novel circulating protein biomarkers, including notch receptor 3 extracellular domain (NOTCH3‑ECD) [154], latent transforming growth factor‑β binding protein 2 (LTBP‑2) [155], the insulin‑like growth factor binding protein (IGFBP) family [153,156], Erb‑B2 receptor tyrosine kinase 3 (ErbB3) [157], and C1q/tumor necrosis factor‑related protein 7 (CTRP7) [158].

#### 3.1.2 Metabolomics

Metabolomic studies have significantly advanced our understanding of PAH by revealing profound metabolic reprogramming. Metabolic profiles can distinguish patients of different risk strata and are closely associated with clinical outcomes [159]. Multiple plasma metabolites are altered in relation to disease severity and prognosis, particularly involving mitochondrial energy metabolism and nucleotide modification pathways [132,160]. Moreover, multi-omics integration has shown that, in addition to mitochondrial energy metabolism abnormalities, disruptions in amino acid metabolism (e.g., the tryptophan–kynurenine pathway), polyamine metabolism, and lipid/sphingolipid metabolism also contribute to PAH pathogenesis [159,160,161,162,163]. Several potential diagnostic and prognostic metabolic biomarkers have emerged, including lipid ratios [164], the dimethylarginine pathway, cysteine, and triacylglycerols (TAGs) [165]. Moreover, metabolomic evidence indicates biological overlap with heart failure with preserved ejection fraction in a subset of PAH patients [166], highlighting shared pathogenic pathways and underscoring metabolomics as a pivotal tool for identifying novel biomarkers and mechanistic insights.

#### 3.1.3 Single-Cell Omics

Single-cell omics have revealed cellular heterogeneity and regulatory networks in PAH pulmonary vasculature. Studies show that endothelial cells, smooth muscle cells, and immune cells exhibit abnormal states in PAH [20,167,168] and play key roles in vascular remodeling. Single-cell analyses have identified characteristic genes such as *SOX17*↓ [169], and *BMPR2*↓ [170], suggesting potential cell-specific biomarkers and signaling networks. Some studies combining animal models with human data [171] further elucidate changes in cell populations related to pulmonary vascular remodeling and propose these as potential therapeutic targets. Building on this, multi-omics studies integrating transcriptomics, GWAS, and single-cell data have constructed regulatory networks and identified potential protective molecules such as Asporin [172], reflecting a shift from single biomarkers to systems biology analyses.

#### 3.1.4 Imaging Techniques

Imaging techniques in PAH primarily reflect right ventricular remodeling and functional changes. Cardiac magnetic resonance (CMR) parameters, such as right ventricular ejection fraction, T1 mapping, and myocardial extracellular volume, can effectively predict clinical worsening and mortality risk [173]. Beyond conventional CMR, positron emission tomography/magnetic resonance imaging (PET/MRI)-detected enhanced right ventricular glucose metabolism is associated with functional decline [174], providing imaging evidence for the relationship between right ventricular metabolic remodeling and functional failure. Additionally, echocardiography, 4D flow MRI, and 3D cardiac ultrasound [175,176] are also used for early diagnosis, treatment monitoring, and risk assessment, emphasizing the importance of multimodal imaging integration for precision clinical management (Table 1, Ref. [151,152,153,154,155,157,158,161,163,164,165,169,170,177,178,179,180,181,182]).

**Table 1.** **Emerging biomarkers in pulmonary arterial hypertension: insights from omics studies**.

Omics category	Biomarker	Outcomes	Ref
Proteomics	IL1R1/ST2, TIMP-1, TIMP-2, plasminogen, ApoE, EPO, complement factor H and factor D, IGFBP-1	Identifies patients with pulmonary arterial hypertension with a high risk of mortality, independent of existing clinical assessments	[151]
	CRIM1, HGF, FSTL3, PLAUR, CLSTN2, SPON1	Independently associated with death/lung transplantation	[152]
	NOTCH3 extracellular domain in serum	Predicting the presence, disease severity, progression and survival of individuals with IPAH	[154]
	LTBP-2	Associated with right ventricular function	[155]
	Insulin-like Growth Factor Binding Protein-2 (IGFBP-2)	Associated with PAH severity and mortality	[153]
	ErbB3	ErbB3 regulates endothelial dysfunction in hypoxia-induced pulmonary hypertension	[157]
	CTRP7	Elevated CTRP7 levels correlate with improved hemodynamic responses to therapy	[158]
	Chemerin	Surrogate biomarker for PVR in SSc-PAH	[177]
Metabolomics	Plasma kynurenine pathway metabolites (e.g., kynurenine, 3-hydroxykynurenine)	KP-metabolites predict response to PAH therapy and survival of PAH patients	[161]
	kynurenine, homoserine, tryptophan, AMP, and spermine	Potential biomarkers for IPAH	[163]
	Lipid Ratios	Provide diagnostic and prognostic information, complementing established markers	[164]
	dimethylarginine pathway, cysteine, and triacylglycerides	Associated with exercise intolerance in PAH	[165]
Single-cell Omics	KLF2, RASIP1, DEPP1	Identifying HTS cells	[182]
	SOX17, E2F1	E2F1 signaling was shown to mediate the SOX17 deficiency-induced EC dysfunction	[169]
	BMPR2, inhibitors of DNA-binding proteins (IDs)	Regulates right ventricular function in CHD-PAH	[170]
	HLA-DPA1	HLA-DPA1 was markedly reduced in NCM of IPAH patients, contributing to immune imbalance and vascular remodeling.	[178]
	KLF2, RASIP1 and DEPP1	Potential drivers of endothelial dysfunction in PAH	[182]
	POSTN, CCDC80	Hub genes up-regulated in rat and human IPAH cohorts	[179]
	CTNNB1, MAPK3, ITGB1, HSP90AA1, DDX5	Promising diagnostic significance for IPAH	[180]
	IGF1, KARS, CASP3, CDKN2A	Associated with fibroblast-mediated glycolysis in IPAH	[181]

Table note:Abbreviations: IL1R1/ST2, interleukin-1 receptor-like 1 (ST2); TIMP-1, tissue inhibitor of metalloproteinases-1; TIMP-2, tissue inhibitor of metalloproteinases-2; ApoE, apolipoprotein E; EPO, erythropoietin; IGFBP-1, insulin-like growth factor binding protein-1; CRIM1, cysteine-rich transmembrane BMP regulator 1; HGF, hepatocyte growth factor; FSTL3, follistatin-like 3; PLAUR, plasminogen activator, urokinase receptor; CLSTN2, calsyntenin-2; SPON1, spondin-1; LTBP-2, latent transforming growth factor beta binding protein-2; IGFBP-2, insulin-like growth factor binding protein-2; CTRP7, C1q/tumor necrosis factor-related protein 7; PVR, pulmonary vascular resistance; SSc-PAH, systemic sclerosis-associated pulmonary arterial hypertension; KP, kynurenine pathway; AMP, adenosine monophosphate; KLF2, Kruppel-like factor 2; RASIP1, Ras-interacting protein 1; DEPP1, decidual protein induced by progesterone 1; SOX17, SRY-box transcription factor 17; E2F1, E2F transcription factor 1; EC, endothelial cell; BMPR2, bone morphogenetic protein receptor type 2; IDs, inhibitors of DNA binding proteins; CHD-PAH, congenital heart disease-associated pulmonary arterial hypertension; HLA-DPA1, major histocompatibility complex, class II, DP alpha 1; NCM, non-classical monocytes; IPAH, idiopathic pulmonary arterial hypertension; POSTN, periostin; CCDC80, coiled-coil domain containing 80; CTNNB1, catenin beta 1; MAPK3, mitogen-activated protein kinase 3; ITGB1, integrin subunit beta 1; HSP90AA1, heat shock protein 90 alpha family class A member 1; DDX5, DEAD-box helicase 5; IGF1, insulin-like growth factor 1; KARS, lysyl-tRNA synthetase; CASP3, caspase-3; CDKN2A, cyclin-dependent kinase inhibitor 2A.

### 3.2 Risk Stratification

Precise risk stratification is crucial for guiding treatment decisions and improving prognosis in patients with PH. Hoeper et al. [183] validated the risk stratification model of the 2015 European Pulmonary Hypertension Guidelines, which divided patients into low-, medium-, and high-risk groups based on variables such as World Health Organization (WHO) functional classification, 6-minute walk distance (6MWD), and cardiac index. In addition to clinical indicators, new biomarkers and imaging parameters were also integrated into the risk assessment. Malnutrition status was also confirmed to be associated with disease severity and poor prognosis in IPAH as assessed by the Controlling Nutritional Status score (CONUT score) [184]. These studies have collectively driven the refinement and personalization of PH risk stratification models.

## 4. Treatment Strategies and Targeted Drug Therapy

### 4.1 Current Treatment Strategies

Despite significant advances in targeted therapies over the past three decades [185], the median survival of patients with PAH remains only around seven years [186]. Mortality in intermediate- and high-risk patients over the short to mid-term remains high. Conventional PAH therapies mainly target three pathways: endothelin-1, nitric oxide (NO), and prostacyclin [13,14,187]. These therapies primarily act by regulating vascular tone. Although these drugs clearly improve symptoms, exercise capacity, and short-term outcomes, they cannot halt disease progression. Long-term survival remains unsatisfactory. Endothelin receptor antagonists, such as Bosentan [188] and Ambrisentan [189], have shown clinical benefits in PAH patients. Compared with placebo, they significantly increase the 6MWD from baseline. Subsequent studies with Macitentan [190] demonstrated consistent efficacy regardless of prior PAH therapy and reduced the occurrence of clinical worsening events. Drugs targeting the NO pathway, including phosphodiesterase type 5 inhibitors (PDE-5i) and soluble guanylate cyclase (sGC) stimulators, enhance NO/cGMP signaling to promote vasodilation and inhibit proliferation. Sildenafil [191] and Tadalafil [192] improved 6MWD after 12 and 16 weeks of treatment, respectively. Similarly, the sGC stimulator Riociguat [193] improved exercise capacity and secondary efficacy endpoints in PAH patients.

### 4.2 The Core Role of Prostacyclin-Based Therapies

In PAH, endothelial dysfunction leads to reduced endogenous prostacyclin (PGI_2_) production [194]. The loss of signaling through the prostacyclin (IP) receptors is considered a key pathological mechanism underlying persistent pulmonary vasoconstriction, smooth muscle proliferation, and thrombosis [195]. Targeting the prostacyclin pathway can therefore not only induce vasodilation and inhibit proliferation [196], but also suppress platelet aggregation and improve pulmonary hemodynamics [197,198]. Clinical trials and recent meta-analyses have confirmed that prostacyclin analogs, such as epoprostenol and treprostinil [199,200], improve 6MWD, hemodynamics, and reduce clinical worsening events in PAH patients. Compared with placebo, the oral prostacyclin receptor agonist selexipag reduces the risk of death and related complications [201,202]. At the molecular level, recent structural biology studies have revealed the cryo-EM structure of the IP receptor bound to active metabolites, such as MRE-269, the active form of selexipag [203]. This provides a molecular basis for understanding ligand selectivity, receptor activation, and G-protein coupling, and supports the development of highly effective IP agonists with low side effects. RNA sequencing has shown that plasma C1q/tumor necrosis factor‑related protein 7 (CTRP7) levels are elevated in PAH patients. CTRP7 may reduce selexipag response by promoting Rab5a-mediated endocytosis of the IP receptor (*PTGIR*) [158], suggesting that CTRP7 inhibition could reverse drug resistance and provide a new avenue for precision combination therapy. Currently, Ralinepag, a next-generation, highly selective IP receptor agonist, has shown, in pivotal phase III trials, a 55% reduction in the risk of the composite endpoint of clinical worsening and death [204]. These highlight Ralinepag as a promising therapeutic agent for clinical application.

### 4.3 Application of Prostacyclin in PH Associated With Lung Diseases

Prostacyclin therapies are expanding beyond WHO group 1 PH to non-group 1 PH (groups 2–5). For example, inhaled treprostinil has been approved for pulmonary hypertension associated with interstitial lung disease (PH-ILD) [200,205]. However, it is associated with local adverse effects such as cough and throat irritation. The novel dry powder inhaler formulation, Yutrepia, may simplify dosing and improve patient adherence [206]. In PH-COPD, the PERFECT study showed a treatment response markedly different from PH-ILD. The study was terminated early due to increased serious adverse events in the treatment group [207]. Although pulmonary vascular resistance improved, respiratory function was not significantly enhanced [208], highlighting the need to identify suitable patient populations in PH of different etiologies. The SELECT study indicated that adding selexipag to standard therapy in inoperable or persistent/recurrent chronic thromboembolic PH (CTEPH) did not significantly reduce pulmonary vascular resistance [209]. Recent data from the CTREPH open-label extension study showed that subcutaneous treprostinil, as part of multimodal therapy, was safe long-term and provided sustained clinical benefits in severe CTEPH patients [210]. The evidence for prostacyclin use in CTEPH remains weaker than in PAH. In summary, prostacyclin therapy remains a cornerstone of PAH treatment and is entering treatment of PH-ILD. However, drug responses vary substantially across different PH etiologies. Future research should focus on optimized formulations, patient subtype selection, and biomarker-guided individualized therapy.

### 4.4 Emerging Targets and Drug Development

#### 4.4.1 *BMP/TGF-β* Pathway-Targeted Drugs

Imbalance of BMP/TGF-β signaling, particularly *BMPR2* downregulation and excessive activation of the Activin-Smad2/3 pathway, is considered a major molecular basis of pulmonary vascular remodeling in PH [211,212,213]. Therefore, restoring the balance between the protective BMP/Smad1/5/8 pathway and the pathogenic TGF-β/Activin pathway has emerged as an important therapeutic strategy in PAH. Sotatercept is the first fusion protein targeting the activin/BMP signaling pathway. It consists of the extracellular domain of human activin receptor type IIA (Act RIIA) fused to the Fc fragment of human IgG1 [67]. Sotatercept acts as a ligand trap for members of the TGF-β superfamily, including Activin A, GDF8, and GDF11. It restores the balance between the pro-proliferative Activin/TGF-β pathway and the anti-proliferative BMP pathway [15,16]. Sotatercept has now been approved for the treatment of adult WHO group 1 PH patients. This marks a transition in PAH therapy from traditional vasodilator-based treatment toward disease-modifying strategies aimed at reversing vascular remodeling. Data from the phase III ZENITH trial demonstrated that, in high-risk PAH patients with WHO functional class III–IV receiving maximally tolerated background therapy, the addition of sotatercept reduced the risk of the primary composite endpoint by 76%. Improvements were also observed in WHO functional class, 6MWD, and hemodynamic parameters [214,215]. Long-term follow-up data from the SOTERIA study further showed sustained reductions in NT-proBNP levels and improvement in risk stratification scores [216]. Stable clinical benefits were observed in both advanced high-risk patients and newly diagnosed intermediate- to high-risk patients [214,217]. However, despite its clear efficacy in improving exercise capacity and pulmonary vascular remodeling, the precise mechanisms underlying sotatercept benefit and the optimal responder population remain unclear [218]. Reported adverse events include epistaxis, telangiectasia, dizziness, hypertension, elevated hemoglobin levels, and thrombocytopenia [15,219]. These findings highlight the need for biomarkers and genetic predictors of efficacy and safety. In addition, some studies found no significant improvement in cardiac output after treatment [220]. Case reports have also suggested a potential risk of pericardial effusion associated with sotatercept, particularly in connective tissue disease-associated PAH (CTD-PAH) [221]. Therefore, its long-term cardiac safety requires further evaluation.

Beyond sotatercept, ligand-trap strategies continue to evolve. KER-012 and HS135 are novel fusion proteins developed based on another receptor, activin receptor type IIB (ActRIIB), and are also designed to modulate the TGF-β/Activin/Nodal signaling pathway [222,223]. A phase II randomized controlled trial of KER-012 in PAH was terminated early because of safety concerns [224]. HS135 remains in the early stages of clinical development [225], and further results are awaited. Another therapeutic strategy focuses on enhancing the protective *BMPR2*-Smad1/5/8 signaling pathway. The calcineurin inhibitor FK506 (tacrolimus) enhances *BMPR2* signaling by releasing FKBP12-mediated suppression of BMP receptors [226]. Clinical studies have demonstrated acceptable safety and tolerability of FK506 in stable PAH patients [227]. Furthermore, the SMAD-specific E3 ubiquitin ligase 1 (SMURF1) inhibitor LTP001 has also been investigated to restore BMP signaling [228], although its efficacy remains at an early exploratory stage [229]. BMP9, a natural high-affinity ligand of BMPR2, has also attracted increasing attention in PAH research. Previous studies demonstrated that BMP9 supplementation reversed pulmonary vascular remodeling in severe occlusive PAH animal models [230], suggesting a potential role in restoring pulmonary vascular homeostasis. However, subsequent studies revealed a dual role of BMP9. Multiple mechanistic studies and animal experiments showed that BMP9 antagonism could also prevent or ameliorate PAH. The underlying mechanisms may involve suppression of C-X-C motif chemokine ligand 12 (CXCL12) release and inhibition of pathological phenotypic switching in pulmonary artery smooth muscle cells [231,232]. These findings suggest that BMP9 signaling may exert distinct biological effects under different pathological conditions. Its effects are likely influenced by inflammatory status, receptor expression patterns, and the local microenvironment. Of note, BMP9 exerts context‑dependent dual effects on EndMT: while it may promote endothelial dysfunction and EndMT [233,234], it can suppress IL‑33‑provoked EndMT under some settings [235]. Since most BMP9 studies remain limited to animal models and current preclinical and clinical evidence is inconsistent, the precise role of BMP9 in PAH remains controversial.

#### 4.4.2 Therapies Targeting Growth Factors—Tyrosine Kinase Inhibitors

The receptor tyrosine kinase (RTK) inhibitor imatinib previously demonstrated improvements in exercise capacity and pulmonary hemodynamics in PAH [236]. However, its clinical application was limited by systemic adverse effects. To reduce systemic toxicity, an inhaled RTK inhibitor, seralutinib, has been developed, in the TORREY trial, seralutinib reduced PVR by 14.3% and showed favorable tolerability [237,238].

#### 4.4.3 Inflammation and Immunity-Targeted Drugs

Inflammation and immune dysregulation play important roles in the pathogenesis and progression of PAH. Among therapies targeting the IL-6 pathway, the IL-6 receptor antagonist tocilizumab was previously investigated in PAH; however, it failed to demonstrate significant improvement in PVR [239]. In contrast, the SATISFY-JP study used AI-based clustering analysis to identify a precision subgroup with high IL-6 expression, where treatment with satralizumab for 24 weeks significantly reduced PVR in these patients [240]. This study provided the first important clinical evidence supporting precision immunotherapy in PAH. Beyond the IL-6 pathway, B-cell depletion therapy has also shown promise. A proof-of-concept study demonstrated that rituximab, used as an adjunctive therapy in systemic sclerosis-associated PAH (SSc-PAH), had acceptable safety and showed a trend toward clinical benefit [241]. Case reports have suggested that rituximab may be effective in systemic lupus erythematosus-associated PAH (SLE-PAH) refractory to conventional therapy [242]. These findings indicate that B-cell depletion therapy may represent a potential rescue strategy for refractory SLE-PAH. Interventions targeting the Interleukin-1 (IL-1) inflammatory pathway have also attracted attention. In a pilot study of PAH patients with right heart failure, the recombinant IL-1 receptor antagonist anakinra demonstrated good feasibility and safety [243]. Overall, immunomodulatory therapies targeting inflammatory and immune-related pathways have shown potential therapeutic value in selected PAH subtypes. However, their long-term efficacy and effects on clinical outcomes require further validation in large randomized controlled trials.

#### 4.4.4 Sex Hormone-Related Drugs

Sex hormone-related pathways are also involved in the pathogenesis and progression of PH. Regarding inhibition of estrogen synthesis, the aromatase inhibitor anastrozole significantly improved 6MWD in postmenopausal women and male patients with PH [244,245], suggesting a potential role in slowing disease progression. However, subsequent larger validation studies found that routine anastrozole treatment did not significantly improve exercise capacity [246], indicating that its clinical efficacy remains controversial. Estrogen receptor antagonism has also been investigated. Tamoxifen showed therapeutic potential in preclinical models of hereditary PAH. In *BMPR2*-mutant mice, tamoxifen partially reversed pulmonary vascular disease (PVD) [247]. Based on these findings, a phase II randomized controlled trial was subsequently initiated. However, results from human studies have not yet been reported [248]. At the same time, dehydroepiandrosterone (DHEA) has also been considered a potential therapeutic agent. Animal studies demonstrated preventive and therapeutic effects of DHEA in chronic hypoxic PH, left-sided heart disease-associated PH, and severe IPAH models [249,250,251]. The underlying mechanisms may involve attenuation of progressive right ventricular ischemia and fibrosis. Furthermore, in a pilot study of COPD-PH, DHEA treatment improved both 6MWD and pulmonary hemodynamics [252]. However, evidence from large randomized controlled trials (RCTs) is still lacking. Beyond sex hormone pathways, mineralocorticoid receptor (MR)-related signaling has emerged as another potential therapeutic target. MR antagonists, including spironolactone and finerenone, not only inhibit the proliferation of PASMCs derived from PAH patients but also attenuate pulmonary vascular remodeling, pulmonary inflammation, and PH severity in rat models [253,254], suggesting multiple protective effects of MR blockade. Overall, therapeutic strategies targeting hormone- and receptor-related pathways have shown promising potential in PH.

#### 4.4.5 Metabolic Pathway-Targeted Drugs

Metabolic abnormalities are considered one of the central pathogenic mechanisms underlying PH. Therefore, therapeutic strategies targeting metabolic reprogramming have emerged as an important area of PH research. For example, the anti-anginal agent ranolazine [255], sodium-glucose cotransporter 2 (SGLT2) inhibitors [256], and other metabolic modulators may provide potential benefits in PH by improving energy metabolism and cardiopulmonary function. In addition, metabolic interventions targeting glucose dysregulation have attracted considerable attention. Preclinical studies in animal models of PAH showed that dichloroacetate (DCA) improves hemodynamic parameters and right ventricular function [257]. Both animal experiments and human studies have demonstrated favorable safety and tolerability of metformin in PAH patients [258,259]. Because metabolic dysfunction in PAH involves multiple interconnected metabolic pathways, combined modulation of different metabolic pathways may be more effective than single-target interventions in reversing pulmonary vascular remodeling [260]. Although current clinical evidence remains limited, existing findings suggest that metabolic pathways may become important therapeutic targets for future PAH treatment.

## 5. Disease Heterogeneity and Research Gaps

Despite significant progress in the field of PH research, there are still many challenges and research gaps that limit a comprehensive understanding and effective intervention of the disease. The clinical phenotypes of PH are complex and diverse, and there is significant heterogeneity in patients’ responses to existing treatments [6,261].

Although some biomarkers and multi-omics approaches have been proposed for diagnosis and prognosis assessment, and even for differentiating different PH subtypes [154,262], there is still a lack of a mature, comprehensive strategy that can guide precise clinical typing and individualized treatment. Especially for the unique phenotype of IPF patients with “no pulmonary hypertension but pulmonary vascular dysfunction” [7], the pathophysiological characteristics and the best management plan remain unclear. How to integrate multi-omics data, imaging features, and clinical information to achieve precise classification of PH, aiming to identify subpopulations with different prognostic and therapeutic responses, is an urgent problem to be solved at present.

## 6. Conclusion

This review provides a comprehensive overview of PH, encompassing its pathological mechanisms, diagnostic strategies, and therapeutic advances. Research has progressed from focusing solely on vascular constriction imbalance to a multi-level, systems-based understanding. Dysregulation of BMPR2 signaling and pulmonary vascular remodeling remain central to disease pathogenesis. Integration of genetic, epigenetic, metabolic, immune, and intercellular network interactions offers a more complete view of disease progression and underpins precision medicine approaches. Multi-omics and single-cell analyses have revealed extensive disease heterogeneity and facilitated discovery of novel biomarkers. Therapeutic strategies are shifting from conventional vasodilators toward disease-modifying interventions targeting the *BMP/TGF-β* pathway, exemplified by sotatercept. Immunomodulatory, hormonal, and metabolic-targeted therapies also show potential, although clinical heterogeneity and long-term safety remain to be fully evaluated. Future research should dissect the complex crosstalk among genes, environment, and cellular networks, while bridging the gap between basic discoveries and clinical translation. Translating molecular targets into individualized therapies holds promise for improving long-term outcomes in PH patients.
